# Optimal Design of PV Systems in Electrical Distribution Networks by Minimizing the Annual Equivalent Operative Costs through the Discrete-Continuous Vortex Search Algorithm

**DOI:** 10.3390/s22030851

**Published:** 2022-01-23

**Authors:** Brandon Cortés-Caicedo, Federico Molina-Martin, Luis Fernando Grisales-Noreña, Oscar Danilo Montoya, Jesus C. Hernández

**Affiliations:** 1 Facultad de Ingeniería, Instituto Tecnológico Metropolitano, Medellín 050036, Colombia; brcortesc@gmail.com; 2 Estudiante de Doctorado, Departamento de Ingeniería Eléctrica, Campus Lagunillas s/n, University of Jaén, Edificio A3, 23071 Jaén, Spain; federicomolinamartin@gmail.com (F.M.-M.); luisgrisales@itm.edu.co (L.F.G.-N.); 3 Department of Electromechanical and Mechatronic, Faculty of Engineering, Instituto Tecnológico Metropolitano, Medellín 050036, Colombia; 4 Facultad de Ingeniería, Universidad Distrital Francisco José de Caldas, Bogotá 110231, Colombia; odmontoyag@udistrital.edu.co; 5 Laboratorio Inteligente de Energía, Universidad Tecnológica de Bolívar, Cartagena 131001, Colombia; 6 Department of Electrical Engineering, Campus Lagunillas s/n, University of Jaén, Edificio A3, 23071 Jaén, Spain

**Keywords:** annual operative cost, discrete-continuous vortex search algorithm, location and sizing of PV systems, AC and DC distribution systems

## Abstract

This paper discusses the minimization of the total annual operative cost for a planning period of 20 years composed by the annualized costs of the energy purchasing at the substation bus summed with the annualized investment costs in photovoltaic (PV) sources, including their maintenance costs in distribution networks based on their optimal siting and sizing. This problem is presented using a mixed-integer nonlinear programming model, which is resolved by applying a master–slave methodology. The master stage, consisting of a discrete-continuous version of the Vortex Search Algorithm (DCVSA), is responsible for providing the optimal locations and sizes for the PV sources—whereas the slave stage employs the Matricial Backward/Forward Power Flow Method, which is used to determine the fitness function value for each individual provided by the master stage. Numerical results in the IEEE 33- and 69-node systems with AC and DC topologies illustrate the efficiency of the proposed approach when compared to the discrete-continuous version of the Chu and Beasley genetic algorithm with the optimal location of three PV sources. All the numerical validations were carried out in the MATLAB programming environment.

## 1. Introduction

With the growth of the world population, there has been a hasty increase in the demand for power to be able to meet basic human needs [1,2,3]. This has led to the exploitation of electrical energy based on fossil fuels, which are non-renewable, and directly impact the environment, generating pollutants which are emitted into the atmosphere and contribute to global warming [4,5,6]. It is for this reason that renewable energy sources were chosen to attempt to supply energy demands; photovoltaic (PV), wind, and hydroelectric generation are three sources widely used in electrical systems due to their clean and unlimited energy [4,7,8]. Similarly, due to the importance that they have acquired in recent years, the use of this type of energy has undergone rapid technological development, ensuring its accessibility around the world at reasonable prices [4]. Note that, in tropical countries, PV systems are the most promising renewable generation technologies, as in Colombia [9].

In recent years, the Ministry of Mines and Energy in Colombia has developed various strategies to promote the use of renewable energy [10]. One of these proposals resulted in Law 1715 of 2014, which aims to promote the development and integration of distributed generation in the electricity system in urban and rural areas [11]. However, since rural areas are difficult to access and the integration of these areas to the National Interconnected System produce exorbitant costs, diesel-dependent sources were decided upon to generate energy [12]. The recent report (September 2021) of the Institute for Planning and Promotion of Energy Solutions for Non-Interconnected Zones (IPSE) presents that diesel generation has a capacity of 267,911 kW, benefiting around 201,412 users all over the Colombian territory [12]. Even if this type of non-renewable resource is frequently used due to its high efficiency and easy attainment in the market, it produces greenhouse gases and other pollutants directly impacting the atmosphere [12]. To combat this issue, solar generation has gained strength in rural areas in Colombia, owing to the rich abundance of solar resources [12]. Currently in the country, 21,710 kW of PV generation has been installed, supplying electricity to about 34,500 users [12]; however, this amounts to only 8.10% of the population who utilize PV generation in rural areas when compared to diesel generation.

The IPSE report reveals that the Caribbean and Pacific regions are the areas with the greatest abundance of solar resources [12]. These areas increase the possibility of integrating PV generation sources in a country like Colombia, which permits the proposal of solutions that are energetically sustainable to meet the demand of even more users, while simultaneously limiting the use of diesel-based generation. Based on the aforementioned opportunity of promoting renewable generation solutions in tropical countries, efficient optimization techniques are required to identify the location and sizing of these systems in distribution networks [13]. To address this problem, this research document proposes an objective function that simultaneously minimizes generation costs and investment, operation, and maintenance costs of PV sources for a planning horizon of 20 years [14]. To confront the aforementioned concern, a master–slave optimization methodology is suggested to solve the mixed-integer nonlinear programming model (MINLP) that represents this problem. Therefore, a continuous-discrete version of the vortex search algorithm, recently proposed by [13], is used to locate and size PV units in electrical distribution grids considering the annualized investment and operating costs along with the studying period.

In the specialized literature, it is possible to find different optimization options that rectify the problem of the location and dimension of the distributed generation in distribution networks. Some of these include Genetic Algorithms [15,16], Particle Swarm Optimization [17], Teaching Learning Based Optimization [18], Population-Based Incremental Learning [19], Vortex Search Algorithm [13], Discrete Sine Cosine Algorithm [20], Technique of Smalling Area [21], Improved Harris Hawks Optimizer [22], mathematical based-approaches in GAMS (i.e., General Algebraic Modeling System) [23,24], and Newton-Based metaheuristic optimizers [14], among others. The main characteristic of the optimization methodologies described above is that they use the master–slave optimization scheme to solve the problem of both location and optimal sizing of the distributed generation through the minimization of power losses for a given demand condition, which does not replicate what happens in reality given that the system loads and generation of renewable energy exhibit dynamic behavior throughout a day of operation [14].

Similar to the metaheuristic optimization methods described above, in this work, a master–slave methodology is considered to solve the problem of PV sources’ location and dimension. The algorithm used in the master stage is the discrete-continuous version of the algorithm of vortex search originally planted in [13] to determine the location and sizes of the PV sources, in conjunction with the backward/forward matrix power flow method in the slave stage to determine the total annual operating costs. However, the main difference with the classical literature approaches is that this research considered the daily expected generation and demand curves, demand expected growth rate, as well as the expected return rate of the investments by part of the utility, among other aspects. It is worth mentioning that, in the specialized literature, there are some documents that have addressed the multi-period problem in distribution networks with renewable energies, some of which include the optimal location and dimension of PV sources in DC systems to minimize greenhouse gas emissions where they solve the MINLP mathematical model using GAMS [25]. The optimal sizing and location of the distributed generation will minimize the energy losses from using a hybrid metaheuristic algorithm [26]. The wind turbines’ location and sizing are considered keeping in mind the possibility of injecting reactive power to minimize energy losses using GAMS and its MINLP tools to solve the mathematical model [27]. The optimal location, sizing, and power factor of the distributed generation sources seek to minimize energy losses using a differential evolution algorithm [28]. While the PV sources’ location and sizing aim to minimize energy losses where they solve the mixed-integer convex model using MATLAB CVX [29]. The optimal placement and sizing of PV sources take into consideration the uncertainties and stochastic nature of the PV generation [30,31]. Authors in [14] presented the application of the recently developed Newton Metaheuristic Algorithm to solve the problem of the optimal placement of PV sources allowing for their investment and operative costs; and authors of [16] proposed the application of the discrete-continuous version of the Chu and Beasley genetic algorithm to solve the same problem. Both of these have been taken as references as they are the only two approaches analyzing the mentioned aspects for the distribution networks in the last few years.

Based on the previous revision of the state of the art regarding dispersed generation inclusion in distribution networks, the main contributions of this research work are listed below:

- The generalization of the proposed master–slave optimization algorithm to accurately locate and size the PV sources in electrical distribution networks with AC or DC operating technologies, which were not previously reported in the current literature.
- The improvement of the current literature reports for the IEEE 33- and 69-bus systems with the classical Chu and Beasley genetic algorithm.

The rest of this document is arranged as follows: Section 2 presents the mathematical representation of the problem of the location and optimal sizing of PV generation units in distribution systems considering the minimization of total annual operating costs in a given planning period; Section 3 showcases the DCVSA incorporating the backward/forward matrix power flow method, while Section 4 describes the main characteristics of the IEEE 33- and 69-bus systems; Section 5 reveals the results obtained for the location and dimension of the PV units in addition to the total annual operating costs of the test systems in both their AC and DC versions. Finally, Section 6 exposes the conclusions and future works extracted from the development of this research article.

## 2. Mathematical Formulation

The problem of the location and optimal sizing of PV systems in distribution networks can be represented by an MINLP model. The binary variables, which are the decision variables of the problem, are related to the location of the PV units. On the other hand, their continuous part is provided by the solution to the power flow formulation, which corresponds to a nonlinear problem given the nature of its equations [32,33]. The complete optimization model will be formulated in the complex domain to simplify the mathematical power flow solution associated with the slave stage [13].

### 2.1. Formulation of the Objective Function

Generally, when there is a dynamic power flow (inclusion of time dependence) together with the integration of PV units to the distribution system, the interest is focused on minimizing the total costs of the purchase of energy in the substation node that connects to the distribution system with the transmission/sub-transmission network [34]. Therefore, the objective function is composed of the annualized costs of purchasing energy at the substation node added with the PV units’ annualized investment and maintenance costs. Each component of the objective function is presented from (1) to (3):

(1) $$\min\;\;A_{cost}=f_{1}+f_{2},$$

(2) $$f_{1}=C_{kWh}T\left(\frac{t_{a}}{1-{\left(1+t_{a}\right)}^{-N_{t}}}\right)real\left(\sum_{h\in H}\sum_{k\in N}s_{k,h}^{cg}\Delta h\right)\left(\sum_{t\in T}{\left(\frac{1+t_{e}}{1+t_{a}}\right)}^{t}\right),$$

(3) $$f_{2}=C_{pv}\left(\frac{t_{a}}{1-{\left(1+t_{a}\right)}^{-N_{t}}}\right)real\left(\sum_{k\in N}s_{k}^{pv}\right)+C_{O\&M}T\;real\left(\sum_{h\in H}\sum_{k\in N}s_{k,h}^{pv}\Delta h\right),$$

where $A_{cost}$ represents the total annual operative costs in the distribution network; $f_{1}$ is the component of the objective function that models the annualized energy purchasing cost in the substation terminals. $f_{2}$ is the component of the objective function regarding the annualized investment and operating costs in PV sources. $C_{kWh}$ refers to the average energy purchasing costs of the energy in the spot market, while *T* corresponds to the number of days in an ordinary year (i.e., 365 days). $t_{a}$ is the internal rate of return expected for the investments made by the distribution company during the duration of the project. $N_{t}$ is the number of periods considered in the planning horizon. $s_{k,h}^{cg}$ indicates the complex power generation in the terminals of the conventional source connected at node *k* during the period *h*. Δh is the duration in which the electrical variables are assumed to be constant. $t_{e}$ is the expected percentage of the increase in the cost of purchasing energy during the planning horizon, whereas $C_{pv}$ represents the average cost of installing one kW of PV generation. $s_{k}^{pv}$ relates the size of a PV source connected at node *k*, and $C_{O\&M}$ represents the maintenance and operating costs of a PV generation unit. $s_{k,h}^{pv}$ corresponds to the complex power generation provided by each PV source connected at node *k* in the period *h*. Observe that N, H, and T are the sets that contain all the nodes of the distribution network, time periods in a daily operation scenario, and number of years of the planning horizon, respectively.

### 2.2. Set of Constraints

The issue of the optimal location and sizing of PV systems in distribution networks has a set of restrictions corresponding to the different operational limitations found in distribution systems, such as voltage regulation limits, power equilibrium at each node, and the devices’ capabilities, among others. The complete list of constraints for the studied problem are listed from (4) to (12):

(4) $$s_{k,h}^{cg}+s_{k,h}^{pv}-S_{k,h}^{d}=v_{k,h}{\left(\sum_{j\in N}Y_{kj}v_{j,h}\right)}^{*},\left\{\begin{array}{cc} \forall k\in N, & \forall h\in H \end{array}\right\},$$

(5) $$s_{k,h}^{pv}=s_{k}^{pv}G_{h}^{pv},\left\{\begin{array}{cc} \forall k\in N, & \forall h\in H \end{array}\right\},$$

(6) $$imag\left(s_{k,h}^{pv}\right)=0,\left\{\begin{array}{cc} \forall k\in N, & \forall h\in H \end{array}\right\},$$

(7) $$s_{k}^{cg,\min}\leq s_{k,h}^{cg}\leq s_{k}^{cg,\max},\left\{\begin{array}{cc} \forall k\in N, & \forall h\in H \end{array}\right\},$$

(8) $$y_{k}s_{k}^{pv,\min}\leq s_{k}^{pv}\leq y_{k}s_{k}^{pv,\max},\left\{\begin{array}{c} \forall k\in N \end{array}\right\},$$

(9) $$v_{k}^{\min}\leq \left|v_{k,h}\right|\leq v_{k}^{\max},\left\{\begin{array}{cc} \forall k\in N, & \forall h\in H \end{array}\right\},$$

(10) $$|i_{kj,h}|\leq i_{kj}^{\max},\left\{\begin{array}{cc} \forall k\in N, & \forall h\in H \end{array}\right\},$$

(11) $$\sum_{k\in N}y_{k}\leq N_{pv}^{ava},$$

(12) $$y_{k}\in \left\{0,1\right\},\left\{\begin{array}{c} \forall k\in N \end{array}\right\},$$

where $S_{k,h}^{d}$ is the complex power demanded at node *k* in the period *h*. $v_{k,h}$ and $v_{j,h}$ represent the complex voltages at nodes *k* and *j* during the period *h*, respectively, while $Y_{kj}$ is the complex admittance that associates nodes *k* and *j*. $G_{h}^{pv}$ is the expected PV generation curve in the zone of influence of the distribution network. $s_{k}^{cg,\min}$ and $s_{k}^{cg,\max}$ are the complex power bounds regarding the conventional generation connected at node *k*. $s_{k}^{pv,\min}$ and $s_{k}^{pv,\max}$ are the complex power bounds related with the PV generation unit connected at node *k*. $i_{kj,h}$ is the complex current flow through the line that connects nodes *k* and *j* during the period *h*. $y_{k}$ is the binary variable regarding the location of PVs in the distribution network at node *k*, indicating that $y_{k}$ = 1 if the PV source is installed or $y_{k}$ = 0 if not. $v_{k}^{\min}$ and $v_{k}^{\max}$ are the admissible voltage regulation bound limits for the whole nodes on the set N. Finally, $N_{pv}^{ava}$ is a constant parameter associated with the maximum number of PV units available for installation throughout the distribution system.

### 2.3. Model Interpretation

The optimization model (1)–(12) is interpreted as the following: Equation (1) defines the objective function of the problem which is the sum between the annual costs of purchasing energy in conventional generators (i.e., substation nodes as defined in Equation (2)), with the annual investment costs of the PV units including their maintenance and operation costs as proposed in Equation (3). The Equality Equation (4) represents the complex power balance in each node of the system for each period of time; it is the most complex restriction that occurs in the examined problem, and since it is nonlinear and not convex, numerical methods must be used to solve it properly. Equation (5) represents the complex power generated in each PV generation unit for each time period. The Equality Equation (6) establishes that only active power injection by the PV generation units will be considered [7]. The inequality constraint (7) deals with the lower and upper bounds of the power outputs in the conventional sources. It (8) is also a box-type constraint that defines the minimum and maximum complex power generation limits in the PV generation units that will be installed along with the distribution network. The inequality box-type constraint (9) defines the lower and upper voltage regulating bounds for all nodes and periods of time of the planning project. Additionally, it (10) defines the maximum allowed current that can flow for each branch of the network at any period of time. It also (11) defines the maximum PV generation units available for installation in the distribution network. Finally, in (12), the binary nature of the decision variable $y_{k}$ is revealed.

Note that one main complication of the MINLP model defined from (1) to (12) corresponds to the combination of binary and continuous variables with nonlinear non-convex constraints, specifically in the case of the complex balances at each node of the network for each period of time [14]. To solve this type of model, the specialized literature recommends the use of the master–slave optimization methods that simplify the problem presented by separating the location and dimension of the PV generation units from the power balance in the distribution network [35]. Consequently, in the next section, we will present a master–slave optimization approach to solve the MINLP model defined from (1) to (12) by combining a DCVSA in the master stage with the backward/forward power flow method in the slave stage.

## 3. Methodology Proposed

To solve the problem of PV sources’ optimal location and sizing in distribution grids with the aim of minimizing the total annualized operative costs regrading energy purchasing at substation terminals along with the investment and operating costs of PV generation units, we propose the application of the discrete-continuous vortex-search algorithm (DCVSA) in the master stage as initially proposed in [13]. To rectify the slave problem, we use the complex version of the backward/forward power flow method utilizing the information regarding the locations and sizes of the PV generation units provided by the master stage [36], for which we will first describe both the master and slave stages in detail.

### 3.1. Slave Stage: Matricial Backward/Forward Power Flow Method

The matricial backward/forward power flow method is a generalization of the classical iterative sweep method for distribution networks which employs a node–branch incidence matrix to represent the system topology [37,38]. In the formulation of this power flow methodology, it is important to note that the node–branch incidence matrix, i.e., $A\in R^{n\times b}$ is generally a rectangular matrix with *n* rows and *b* columns, *n* being the number of nodes, and *b* the number of branches of the network [39,40]. Additionally, for this matrix, it is assumed that the current flows are arbitrarily selected for all the network’s branches. The node–branch matrix (A) can be built as follows:

- $A_{kl}=1,$ if the current through the line *l* leaves the node *k*;
- $A_{kl}=-1,$ if the current through the line *l* arrives the node *k*;
- $A_{kl}=0,$ if the line *l* is not connected to the node *k*.

By means of the incidence matrix, it is possible to define the voltage drop in the network sections of the system, i.e., $E\in R^{b\times 1}$, as a function of the nodal voltages, i.e., $V\in R^{n\times 1}$, as defined in Equation (13):

(13) $$E=A^{T}V$$

Now, rewriting (13) in terms of the voltage in the conventional sources and the voltage in the demand nodes, the following result is reached:

(14) $$E=A_{s}^{T}V_{s}+A_{d}^{T}V_{d}$$

where $A_{s}\in R^{1\times b}$ is the first row of the incidence matrix which corresponds to the component associated with the node slack. $V_{s}\in R^{1\times 1}$ is the vector that defines the voltage output in the slack node which is assumed to be constant and well-known in power flow studies [36]. $A_{d}\in R^{(n-1)\times b}$ contains the rest of the rows of the incidence matrix and is the component that associates the demand nodes with each other. Finally, $V_{d}\in R^{(n-1)\times 1}$ is the vector containing the variables of interest, i.e., the demanded voltage profiles.

On the other hand, applying Kirchhoff’s first Law for each node of the system, assuming that the demanded currents, i.e., $I_{d}\in R^{(n-1)\times 1}$, leave from each node (negative sign), it is possible to define the relation between the nodal and branch currents (i.e., $J\in R^{b\times 1}$) as defined in Equation (15):

(15) $$\left[\begin{array}{c} I_{s}\\ I_{d} \end{array}\right]=\left[\begin{array}{c} A_{s}\\ A_{d} \end{array}\right]J$$

where $I_{s}\in R^{1\times 1}$ is the vector that contains the net injected current in the slack node.

Additionally, it is possible to relate the voltage drop in the network sections with the current flowing through them by applying Ohm’s Law, as shown in (16):

(16) $$J=Y_{p}E$$

where $Y_{p}\in R^{b\times b}$ is the primitive admittance matrix that contains the inverse of the impedance of each line in its diagonal. Note that, if we replace (14) in (16), and it is also considered the second row of (15), then, the result defined in Equation (17) is reached:

(17) $$I_{d}=A_{d}Y_{p}A_{s}^{T}V_{s}+A_{d}Y_{p}A_{d}^{T}V_{d}$$

Now, if the Tellegen’s theorem is applied [41], then, it is possible to obtain the relationship between the nodal voltage and net current injected into the nodes of the distribution system, i.e., $I\in R^{n\times 1}$, as depicted in (18):

(18) $$S=\mathrm{diag}\left(V\right)I^{*}\Leftrightarrow S^{*}=\mathrm{diag}\left(V^{*}\right)I$$

where $S\in R^{n\times 1}$ is the vector with all the complex power generation at each node of the system. Observe that, if rewritten (18) in terms of the generation and demand, the result in Equation (19) is reached:

(19) $$\left[\begin{array}{c} S_{s}^{*}\\ -S_{d}^{*} \end{array}\right]=\left[\begin{array}{ccc} \mathrm{diag}(V_{s}^{*}) & \; & 0\\ 0 & \; & \mathrm{diag}(V_{d}^{*}) \end{array}\right]\left[\begin{array}{c} I_{s}\\ I_{d} \end{array}\right]$$

where $S_{s}^{*}\in R^{1\times 1}$ y $S_{d}^{*}\in R^{(n-1)\times 1}$ are the complex power generation at the slack node and complex power consumption in the demand nodes, respectively. Now, if we replace (19) in (17) and its obtained and expression for $V_{d}$, the result in Equation (20) yields the following:

(20) $$V_{d}=-Z_{dd}\left[{\mathrm{diag}}^{-1}\left(V_{d}^{*}\right)S_{d}^{*}+Y_{ds}V_{s}\right]$$

where $Z_{dd}$ was defined as ${\left[A_{d}Y_{p}A_{d}^{T}\right]}^{-1}$, and $Y_{ds}$ is also defined as $\left[A_{d}Y_{p}A_{s}^{T}\right]$.

To solve Equation (20), we must add *t* to an iterative counter to determine the final values of the demanded voltages from an initial point. The starting point is usually chosen as the voltage output in the slack node, i.e., $V_{d}^{t}=1_{d}V_{s}$. Thus, the power flow Equation (20) is resolved recursively as defined in (21):

(21) $$V_{d}^{t+1}=-Z_{dd}\left[{\mathrm{diag}}^{-1}\left(V_{d}^{t,*}\right)S_{d}^{*}+Y_{ds}V_{s}\right]$$

The iterative process to solve (21) ends when the convergence criterion is met, as shown in (22):

(22) $$max\left\{\begin{array}{c} \left|\right|V_{d}^{t+1}\left|-\right|V_{d}^{t}\left|\right| \end{array}\right\}\leq \epsilon$$

where ϵ is the maximum admissible error between two consecutive voltage iterations. Here, as recommended in [27], we take a value of $1\times 10^{-10}$ for the parameter ϵ.

**Remark** **1.** *The convergence of the backward/forward matrix method has been demonstrated using the characteristics of the incidence matrix by applying Banach’s fixed point theorem [36].*

Now, we are interested in extending the approach made to the backward/forward matrix power flow method to solve the complex power balance raised in (4), and, thus, we arrive at the recursive formula presented in (23):

(23) $$V_{d,h}^{t+1}=-Z_{dd}\left[{\mathrm{diag}}^{-1}\left(V_{d,h}^{t,*}\right)\left(S_{d,h}^{*}-S_{pv,h}^{*}\right)+Y_{ds}V_{s,h}\right]$$

where $S_{pv,h}^{*}\in R^{(n-1)\times 1}$ is the vector that contains all the complex power generation outputs in the PV generation units at each period *h*. Here, it is worth mentioning that the master stage is entrusted with providing the values of the $S_{pv}^{*}$, contained at each individual of the candidate solutions in the population to the power flow formulation in order to determine energy losses’ value. Similarly, the solution of (23) is obtained when the convergence criterion established in (22) is fulfilled by extending it to the temporal domain, i.e., $\max\left\{\begin{array}{c} \left|\right|V_{d,h}^{t+1}\left|-\right|V_{d,h}^{t}\left|\right| \end{array}\right\}\leq \epsilon$.

An additional important calculation obtained after solving the multiperiod power flow problem corresponds to the component of the objective function related with the total energy purchasing costs at the substation bus. To calculate this, it is necessary to know the value of the complex power output on this node for each period of time, i.e., $S_{cg,h}\in R^{1\times 1}$. To do so, if we replace the demanded voltages obtained in the power flow solution in (16) while also considering the first row of (15), then, the following result yields:

(24) $$I_{s,h}=A_{s}Y_{p}A_{s}^{T}V_{s,h}+A_{s}Y_{p}A_{d}^{T}V_{d,h}$$

which also, when combined with (19), produced the result defined in (25).

(25) $$S_{cg,h}^{*}=\mathrm{diag}\left(V_{s,h}^{*}\right)\left[A_{s}Y_{p}A_{s}^{T}V_{s,h}+A_{s}Y_{p}A_{d}^{T}V_{d,h}\right]$$

Once the power flow in the complex domain has been solved for each time period, as shown in (23), and the complex power generated by the slack node for each time period has been determined, as shown in (25), the fitness function (an adaptation of the objective function common in metaheuristics [42,43]) is then calculated for each individual from the set of candidate solutions resulting in the master stage. The main advantage of using a fitness function instead of the original objective function is that it aids the proposed optimizer in exploring unfeasible regions in search of global optimal solutions in the promissory and unexplored feasible areas of the solution space [44,45]. Furthermore, when the solution space is feasible, then, the fitness function and original objective function take the same numerical value. The proposed fitness function is defined in Equation (26).

(26) $$\begin{array}{cc} F_{f}= & A_{cost}+\alpha_{1}\;\max_{h}\left\{0,|V_{d,h}|-v^{\max}\right\}-\alpha_{2}\;\min_{h}\left\{0,|V_{d,h}|-v^{\min}\right\}\\ \; & -\alpha_{3}\;\min_{h}\left\{0,\mathrm{real}(S_{cg,h}-s_{cg}^{\min})\right\}+\alpha_{4}\;\max_{h}\left\{0,|J_{b,h}|-i^{\max}\right\} \end{array}$$

where $\alpha_{1}$ and $\alpha_{2}$ are penalization factors associated with the violation of the voltage regulation bounds; $\alpha_{3}$ is a penalty factor related with the possible negative power generations in the slack source; and $\alpha_{4}$ indicates the penalization factor regarding the violation of the thermal bounds in all the network branches.

It is worth clarifying that the maximum and minimum limits of the PV generation units’ complex power generation are always followed by the coding proposed by the DCVSA [14]. Conversely, the complex power upper limit on node slack is not taken into account as it is assumed to have enough capacity to support all demands, even if no PV sources are installed.

Algorithm 1 illustrates the implementation of the backward/forward power flow problem to evaluate the fitness function value.

### 3.2. Master Stage: DCVSA

To address the optimal siting and sizing issue of the PV generation units in distribution networks, the DCVSA is employed in this research. The main advantage of a discrete-continuous codification is that the location and sizing problems are solved with a unified representation, which allows the exploration and exploitation of the solution space in an efficient manner by reducing a slave stage, which is the typically known optimal power flow solution in the problems of the optimal placement and sizing of dispersed generation [46].

The structure of the codification to represent the configuration of an *m* individual at the iteration *t* is presented below:

(27) $$P_{m}^{t}=\left[\begin{array}{ccccccccc} 2, & c, & ..., & n & | & p_{2}^{pv}, & p_{c}^{pv}, & ..., & p_{n}^{pv} \end{array}\right];m=1,2,...,N_{i},$$

where $P_{m}^{t}$ represents the configuration of the *m* individual in the set of candidate solutions at the iteration *t* the dimension of which is $1\times 2N_{pv}^{ava}$, *c* being a random integer number that corresponds to a node of the network. $N_{i}$ is the number of individuals in the population. As seen in Equation (27), a solution individual *m* is divided in two components, where the first $N_{pv}^{ava}$ is associated with all the nodes where the PV generation units will be installed (integer part of the codification), whereas the second part of the codification vector defines the optimal sizes of the PV sources, i.e., this part is the continuous one in the codification.

The optimization algorithm used in this research document should be able to define the best set of candidate solutions maintaining the structure displayed in (27) by generating individuals around the best current solution using a Gaussian Distribution in each iteration [47].
**Algorithm 1:** Solution of the multiperiod power flow problem using the matricial backward/forward power flow formulation to determine the fitness function of the studied optimization problem
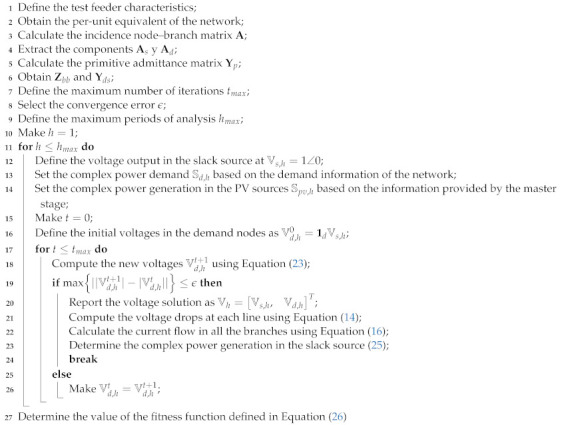


#### 3.2.1. Initial Solution

The vortex search algorithm explores and exploits the solution space with concentric hyper-ellipses [47], where the largest hyper-ellipse radius defines the size of the whole solution space; and its center defines the best current solution. Considering the structure of a solution individual presented in (27), the initial center of the hyper-ellipse is calculated as presented in (28):

(28) $$\mu^{0}=\left(\frac{x_{1}^{\max}+x_{1}^{\min}}{2},\frac{x_{2}^{\max}+x_{2}^{\min}}{2}\right)$$

where $x_{1}^{\min}\in R^{N_{pv}^{ava}\times 1}$ and $x_{1}^{\max}\in R^{N_{pv}^{ava}\times 1}$ are the lower and upper bounds of the decision variables in its discrete components (set of demand nodes where the PV generation units will be installed); $x_{2}^{\min}\in R^{N_{pv}^{ava}\times 1}$ and $x_{2}^{\max}\in R^{N_{pv}^{ava}\times 1}$ are the lower and upper limits of the decision variables regarding the sizes of the PV generation units.

#### 3.2.2. Candidate Solutions

Each individual solution $P_{m}^{t}$ in the set of candidate solution defined as $P^{t}$ is generated with a randomly controlled process through a normal Gaussian distribution around the center of the hyper-ellipse, i.e., μ [48]. The generation of this set of candidate solutions is presented in Equation (29):

(29) $$P_{m}^{t}=p\left(x|\mu ,\Sigma \right)=\frac{1}{\sqrt{{\left(2\pi \right)}^{2N_{pv}^{ava}}\left|\Sigma |\right)}}\exp\left\{-\frac{1}{2}{\left(x-\mu \right)}^{T}\Sigma^{-1}\left(x-\mu \right)\right\},$$

where $x\in R^{2N_{pv}^{ava}\times 1}$ is a vector of random variables, and $\Sigma \in R^{2N_{pv}^{ava}\times 2N_{pv}^{ava}}$ is known as the co-variance matrix. Note that, if Σ has in its diagonal equal values (i.e., the same variance), and if the non-diagonal components are zero, then, the Gaussian distribution will generate hyper-spheres around the solution space. Taking into account these characteristics on the co-variance matrix, it is possible to easily calculate it as presented below [47]:

(30) $$\Sigma =\sigma^{2}I,$$

where σ is the Gaussian distribution variance, and $I\in R^{2N_{pv}^{ava}\times 2N_{pv}^{ava}}$ is an identity matrix with proper dimensions. With (31), the initial standard deviation of the Gaussian Distribution is calculated, and complying in the same way with the coding proposed in (27), we arrive at the following:

(31) $$\sigma^{0}=\frac{\max\left\{x_{1}^{\max},x_{2}^{\max}\right\}-\min\left\{x_{1}^{\min},x_{2}^{\min}\right\}}{2},$$

where $\sigma^{0}$ is also known as the initial *d*-dimensional radius $r^{0}$ of the hyper-ellipse [47].

#### 3.2.3. Updating of the Current Solution

For the solutions to be feasible, it must be guaranteed that the individuals generated and contained in the set of candidate solutions $P^{t}$ are within the limits of the solution space, previously defined for the calculation of the initial center $\mu_{0}$ of the hyper-ellipse. In this sense, the lower and upper limits of the discrete and continuous parts of each individual are verified separately, as depicted in (32) and (33), respectively:

(32) $$P_{dis,m}^{t}=\left\{\begin{array}{cc} P_{dis,m}^{t} & x_{1}^{\min}\leq P_{dis,m}^{t}\leq x_{1}^{\max}\\ x_{1}^{\min}+rand\cdot \left(x_{1}^{\max}-x_{1}^{\min}\right) & \mathrm{otherwise} \end{array}\right.$$

(33) $$P_{con,m}^{t}=\left\{\begin{array}{cc} P_{con,m}^{t} & x_{2}^{\min}\leq P_{con,m}^{t}\leq x_{2}^{\max}\\ x_{2}^{\min}+rand\cdot \left(x_{2}^{\max}-x_{2}^{\min}\right) & \mathrm{otherwise} \end{array}\right.$$

where rand provides random numbers with normal distribution between 0 and 1. Once the lower and upper limits of the discrete and continuous parts of the individuals have been verified, and the ones who were not feasible have been adjusted, the fitness function exhibited in (26) is evaluated for each one. The individual in the set of candidate solutions $P^{t}$ with the best current solutions ($P_{best}$) will be selected as the new center of the hyper-ellipse if, and only if, its fitness function value is better than the previous center $\mu^{t}$. This evolution step is defined through Equation (34):

(34) $$\mu^{t+1}=\left\{\begin{array}{cc} P_{best} & \mathrm{Si}\;F_{f}\left(P_{best}\right)<F_{f}\left(\mu^{t}\right)\\ \mu^{t} & \mathrm{otherwise} \end{array}\right.$$

#### 3.2.4. Radius Reduction

Quite possibly the most important process of the vortex search algorithm is the adaptive adjustment of the radius of the hyper-ellipse; it decreases to zero as the algorithm progresses, which implies that, at the end of the process, the final center of this hyper-ellipse corresponds to the optimal solution of the studied optimization problem. As recommended in [47], an efficient way to reduce the radius of the hyper-ellipse is by using the incomplete inverse Gamma function, which is defined in (35):

(35) $$r^{t}=\sigma^{0}\gamma^{-1}\left(w,a^{t}\right)$$

The incomplete inverse Gamma function can be easily calculated in MATLAB using the command $\mathrm{gammaincinv}(w,a^{t})$ as presented in Equation (36):

(36) $$r^{t}=\sigma^{0}\frac{1}{w}\mathrm{gammaincinv}\left(w,a^{t}\right),$$

where $a^{t}$ is a parameter defined as $a^{t}=1-\frac{t}{t_{\max}}$. Additionally, *w* is defined as $\frac{1}{10}$ following the recommendation in [47].

Finally, a new set of candidate solutions $P^{t+1}$ is generated around the new center considering the new radius defined in Equation (35).

**Figure 1:**
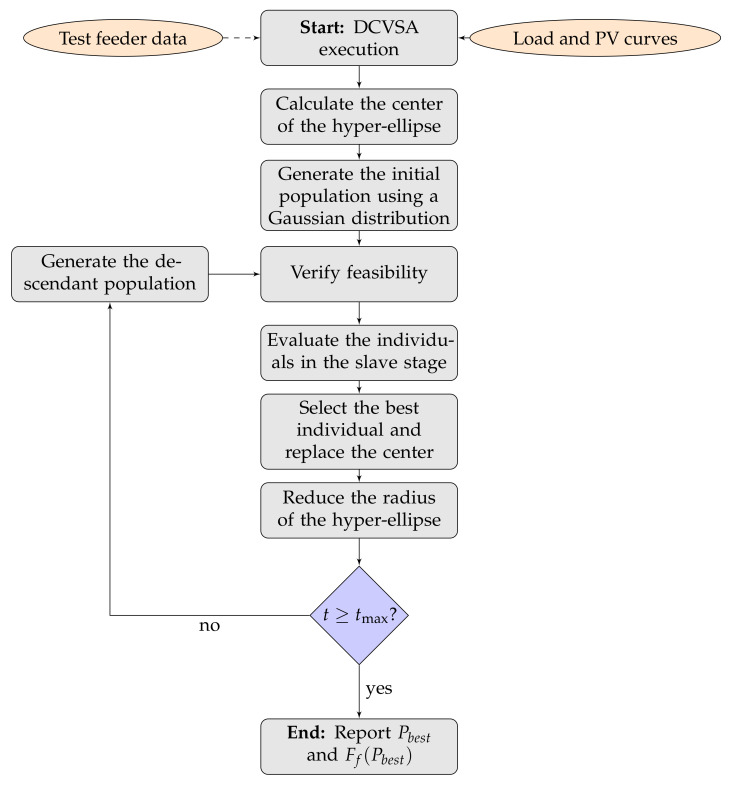
General implementation of DCVSA to solve optimization problems in distribution systems.

The diagram presented in Figure 1 generally summarizes the logic of the DCVSA proposed to define the location and optimal sizing of PV generation units in distribution systems.

## 4. Test Systems

In this section, we present the main characteristics of the test feeder used to validate the proposed master–slave optimizer to locate and size PV generation units in distribution networks. The test feeders considered are composed of 33 and 69 buses, both with radial structure [23].

### 4.1. IEEE 33-Node Test Feeder

**Figure 2:**
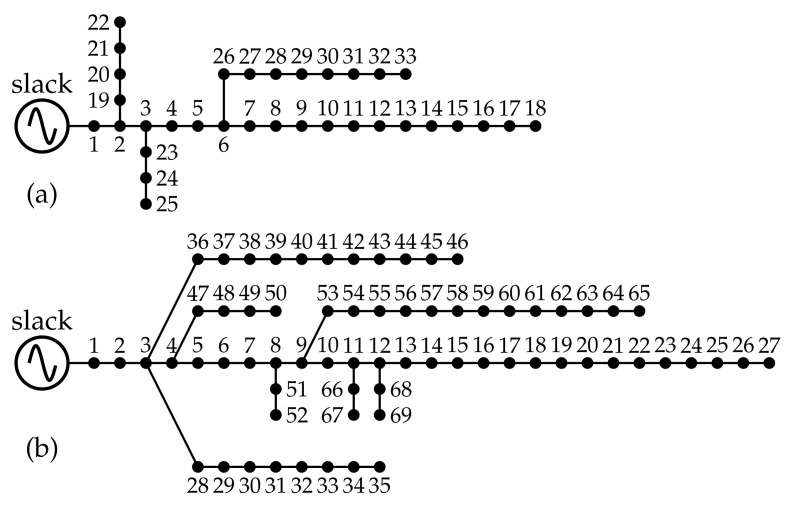
Electrical configuration of the test feeders: (**a**) IEEE 33-node system and (**b**) IEEE 69-bus system.

This test feeder, composed of 32 distribution lines and 33 buses and located at bus 1 in the substation, is operated with 12.66 kV of nominal voltage. It has a total power consumption of $\left(3715+j2300\right)\;$kVA during the peak load scenario. Its electrical configuration is depicted in Figure 2, while its parametric configuration is specified in [49].

### 4.2. IEEE 69-Node Test Feeder

The IEEE 69-bus system is a radial distribution network operated in the substation bus at node 1 with 12.66 kV with 69 nodes and 68 distribution lines (Figure 2 presents the electrical connection among nodes in this distribution grid). During the peek load condition, the total power consumption of this system is $\left(3890.7+j2693.6\right)$ kVA. The complete information regarding lines and powers at each node can be found in [49].

### 4.3. Demand and Generation Curves

**Figure 3:**
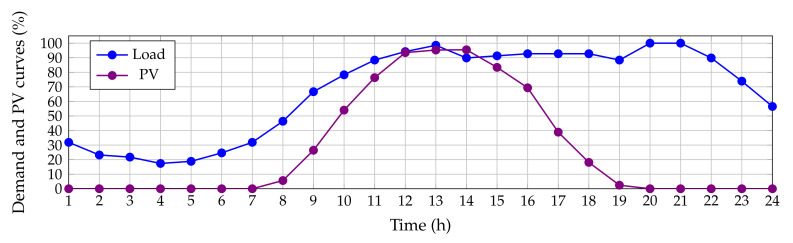
Typical behavior of generation and demand curves for a period of study of 24 h.

To evaluate the impact of the integration of PV generation units in the test systems shown above, the typical Colombian generation and demand curves, shown in Figure 3, are used. Data on the percentage variation in consumption and generation can be found in [50]. According to Figure 3, the peak demand occurs in the hours 20 and 21, while the PV generation units will be able to access the solar resource from 7 to 20 h.

To determine the value of the objective function defined in Equation (1), the parametric information listed in Table 1 is considered. This information was constructed with the data available in [51,52].

## 5. Numerical Results and Simulations

This section presents the numerical validation of the methodology developed to solve the problem of location and optimal sizing of PV generation units in both test feeders under analysis. As a comparative methodology has been employed, the classical Chu and Beasley genetic algorithm using the same discrete-continuous codification is presented in Equation (27). This algorithm was recently proposed in [16] and its acronym is DCCBGA. Moreover, the exact optimization model is also solved using the GAMS optimization package [14]. In the case of the proposed DCVSA, in all the numerical simulations, we use 10 individuals, 1000 iterations, and 100 consecutive evaluations.

In the numerical validations, the following simulation scenarios are proposed:

i. Application of the DCVSA developed and its comparisons with existing methodologies into the IEEE 33- and IEEE 69-node test systems with their AC versions.
ii. The minimization of the total annual operating cost using the proposed master–slave methodology for the DC versions of the IEEE 33- and IEEE 69-bus systems.

The MINLP model of the studied problem defined from (1) to (12) has been implemented and solved in the MATLAB software 2029b using our scripts on a personal computer MD Ryzen 7 3700U (AMD, Santa Clara, CA, USA), 2.3 GHz, 16 GB RAM with 64-bits Windows 10 Home Single Language.

### 5.1. Case 1: Results in the AC IEEE 33-Bus System

Table 2 lists the numerical results obtained after applying the DCVSA to the IEEE 33-bus system considering its AC version. The proposed method is compared with the GAMS results which are gained from the BONMIN solver as well as with the DCCBGA [16]. The numerical results in this table specify the following: the solution provided by the proposed approach finds a better near-optimal result with an additional improvement of US$170.58 per year of operation when compared to the DCCBGA solution. The solution provided by the DCVSA selects nodes 11, 14, and 31 to locate the PV generation units with a global installed capacity of 3648.74 kWp. This generation capability is about 13.33 kWp higher than the solution provided by the DCCBGA. Even if the additional size of the PV sources reached with the DCVSA will increase the investment and operating cost in these renewable energy resources by about US$/year 1682.31, these increments will be compensated with the additional reduction of about US$1852.89 in the energy purchasing costs in the substation bus with respect to the best solution reported by the DCCBGA.

**Figure 4:**
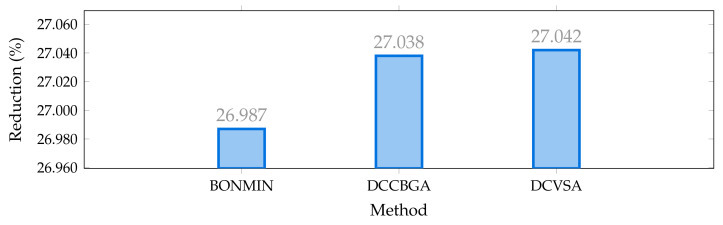
Percentage of reduction of the total grid operative costs in the IEEE 33-bus system in its AC version.

In Figure 4, it is possible to observe the reduction of the total annual operating costs of the IEEE 33-nodes system in percentage terms, methodologies used by [16], and master–slave methodology proposed in this document, with respect to the case base. Note that all the optimization methodologies studied allows an improvement higher than 26% with respect to the benchmark case; nevertheless, the improvement of the DCVSA was about 27.04%, i.e., US$1,000,693.67 per year of operation, which corresponds to an improvement of 0.004% with respect to the solution reported by the DCCBGA. Even if this improvement corresponds to a small reduction regarding the total operating costs of the system, the methodology proposed in this document finds a better optimal solution for the IEEE 33-bus test system. Therefore, this new solution will serve as a reference point for future approaches that may be proposed to solve the problem of PV units’ optimal location and sizing in distribution systems.

To validate the effectiveness and robustness of DCVSA in solving the problem raised in this research document, 100 consecutive evaluations of the master–slave methodology were executed in the IEEE 33-node system, the results of which are exhibited in Table 3. These results present that the DCVSA returns better outcomes when compared with other methodologies that solve the problem of optimal location and dimension of PV generation units in the IEEE 33-node test system. The best response of the DCVSA shows an improvement of 0.006%, i.e., US$170.58 per year of operation when compared to DCCBGA and 0.0764%, i.e., US$2062.43 per year of operation when compared to BONMIN. Similarly, in terms of the average value and worst solutions, the DCVSA reveals an improvement of about US$266.63 and US$517.23 per year of operation compared to the DCCBGA values. This demonstrates the superiority of the proposed methodology to obtain the solution of the aforementioned problem with respect to the best, average, and worst values of the objective function when compared with the DCCBGA.

Regarding the standard deviation, the DCVSA gains a value of US$1154.08 per year of operation, as presented in Table 3, which represents 0.0427% of the variation when compared to the average value, depicting an improvement of approximately US$67.69 per year when compared to the standard deviation of the DCCBGA. This confirms the repeatability properties of the DCVSA to solve the problem raised in this research document because, if it is executed multiple times, for the IEEE 33-node system, it is likely that the proposed methodology will generate the best average response or an answer close to this value.

**Figure 5:**
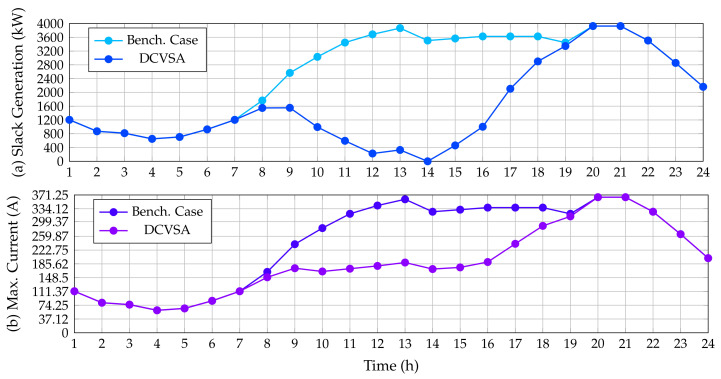
Impact of the PV inclusion in the IEEE 33-bus system: (**a**) power injections in the slack source, and (**b**) maximum current performance.

To establish that the optimal solution provided by the DCVSA fulfills the electrical constraints defined from (4) to (12), which were considered in the fitness function formulation (see Equation (26)), Figure 5 displays a comparison between the power outputs in the slack source for the benchmark case and proposed approach, as well as the contrast in the maximum current of the network before and after installing the PV generation units provided by the DCVSA.

As was expected, the power generation in the slack source tends to follow the behavior of the total active power demand curve in the substation terminals for the benchmark case, which takes place owing to the fact that the slack source is the unique power source activated along with the grid which must support the total grid demand including the electrical power losses. Nonetheless, in Figure 5, we observe that the behavior of the slack active power generation is considerably reduced in the periods of time when the PV generation installed increases their power outputs. Note that, in period 14, when the generation of the PV sources is maximum, the slack generation is zero, thus confirming that restrictions imposed on the slack generation regarding negative power outputs are always fulfilled.

Similarly, Figure 5 depicts the behavior of the maximum current in the IEEE 33-bus system before and after installing the PV generation units. The maximum current for this system always appears in the branch that connects nodes 1 and 2 (the first line from the substation bus). As predicted in the benchmark case, this current follows the demand curve and reaches its maximum value in the periods 20 and 21 (peak demand condition) with a value of 365.2524 A. On the other hand, after the integration of the PV units supplied by the DCVSA, the maximum current that circulates through the system is presented in the network section 1–2 for all time periods; notwithstanding, the current does not follow the same behavior as the power generated at the substation terminals in this case. This is due to the fact that the power injection by the PV units only modifies the active power flow in the AC version of the IEEE 33-bus system, reducing the current circulating through this section of the network, but it does not reach the value of zero as the reactive power flow is the same as in the base case. Likewise, from Figure 5, we see that, with the integration of PV units, the maximum current flowing through the system is less than or equal to the maximum current flowing through the system in the benchmark case, achieving the maximum value of current at hours 20 and 21, with a value of 365.2524 A, which is equal to the current found for the base case because currently, the PV generation units do not have solar resource availability.

**Figure 6:**
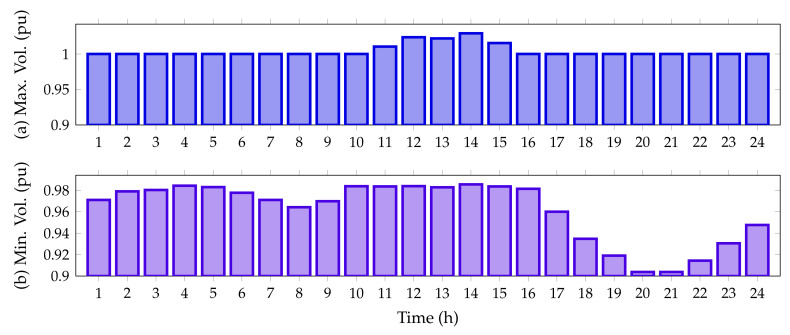
Voltage behavior during the day for the IEEE 33-bus system: (**a**) maximum voltage magnitude, and (**b**) minimum voltage magnitude.

Finally, Figure 6 reveals the maximum and minimum voltage behaviors in the IEEE 33-bus system when PV generation sources are integrated.

From Figure 6, it is possible to perceive that the voltage for all time periods satisfies the voltage regulation constraint being within ±10%. Additionally, from hour 11 to hour 15, when the PV units inject more than 70% of their capacity, the voltage at node 14 exceeds the voltage at the slack node, reaching a maximum value of 1.0291 pu; while the minimum voltage value, as expected, is found at node 18, at hours 20 and 21, with a value of 0.9038 pu (no PV generation available).

### 5.2. Case 1: Results in the AC IEEE 69-Nodes System

Table 4 details the numerical results reached by the proposed and comparative methods in the IEEE 69-node test feeder, including the ones for the benchmark case. From this, it is possible to note that the proposed DCVSA finds a better near-optimal solution with an additional improvement of about US$/year 521.76 with regard to the best solution obtained with the DCCBGA. The solution obtained by the DCVSA selects nodes 16, 61, and 63 to locate three PV generation units with a total installed capacity of 3828.46 kWp. This result implies that the DCVSA installs about 23.33 kWp of additional power when compared with the DCCBGA solution. This additional power implies an increment of US$2946.13 per year of operation in the total investment and operating costs in PV generation units; however, this additional investment allows a reduction in the total grid energy purchasing cost of about US$/year 3467.91 when compared to the DCCBGA, which clearly justifies the additional investment in PV sources.

**Figure 7:**
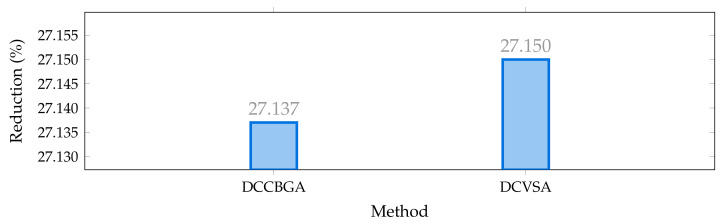
Percentage of reduction of the total grid operative costs in the IEEE 69-bus system in its AC version.

In contrast, Figure 7 outlines the total reduction of the grid operative costs reached by both the proposed and comparative methods when compared to the benchmark case. Note that the DCCBGA and DCVSA allows reductions of more than 27% of the total network costs; nonetheless, the DCVSA solution allows a reduction of 27.15%, i.e., US$/year 1,052,938.37, which represents an additional 0.01345% of improvement to the solution obtained by the DCCBGA.

To validate the effectiveness and robustness of the proposed optimization approach, 100 consecutive evaluations of the whole optimization strategy were made in the IEEE 69-bus system. Numerical results obtained from these evaluations are reported in Table 5. The results in this table proves that the DCVSA depicts a general improvement of the objective function of 0.0185% (i.e., US$521.76 per year of operation when compared with the DCCBGA). Regarding the average and worst solutions, it demonstrates a general improvement of US$/year 458.64 and US$/year 10,318.58 when compared with the same data of the DCCBGA. This verifies the superiority of the proposed methodology to solve the problem of location and optimal sizing of PV generation units in the IEEE 69-bus system, with respect to the best, average, and worst values of the objective function of total annual operation costs in comparison to the classical DCCBGA.

With regard to the standard deviation, the proposed DCVSA has a value of US$/year 2666.56 (see Table 5), which corresponds to a variation of 0.0943% concerning the average value; however, this standard deviation is at least US$/year 160.62 better than the same value reached with the DCCBGA. This demonstrates the repeatability properties of the DCVSA to solve the problem raised in this research document; if it is executed multiple times for the IEEE 69-bus system, it is likely that the proposed methodology will generate the best average response or an answer close to this value.

**Figure 8:**
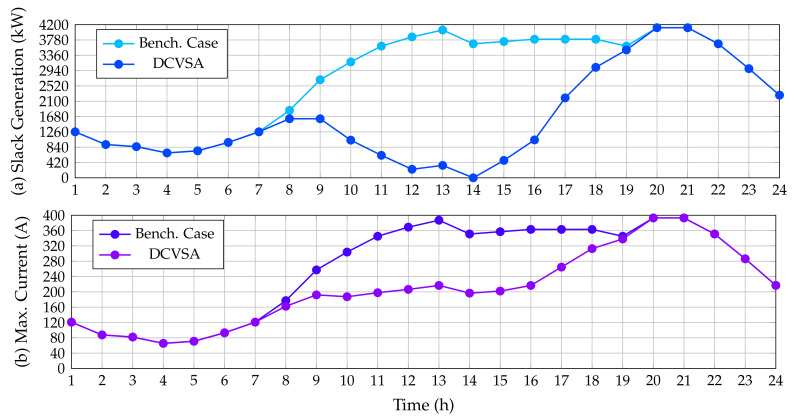
Impact of the PV inclusion in the IEEE 69-bus system: (**a**) power injections in the slack source, and (**b**) maximum current performance.

Figure 8 compares the active power generation in the slack source for the benchmark case and the solution obtained with the proposed DCVSA, as well as places in contrast to the maximum current of the system for the same cases of simulation. As expected, the behavior of the active power generation in this source follows the same performance reported by the IEEE 33-node test feeder. Regarding the current behavior, it is possible to observe that the maximum value for this variable is equal in both simulation cases, since the peak demand consumption appears in hours 20 and 21. The maximum current for this system is in the branch that connects nodes 1 and 2 with a value of 393.0195 A.

**Figure 9:**
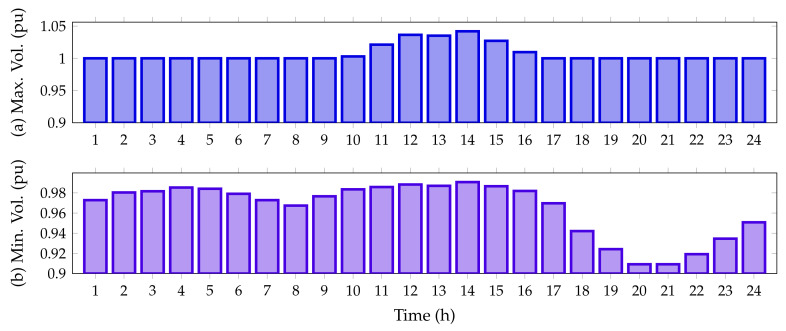
Voltage behavior during the day for the IEEE 69-bus system: (**a**) maximum voltage magnitude and (**b**) minimum voltage magnitude.

Finally, Figure 9 reveals the maximum and minimum voltage behaviors in the IEEE 69-bus system when integrated with the PV generation sources.

From Figure 6, we note that the voltage for all time periods satisfies the voltage regulation constraint as it is within ±10%. Furthermore, from hour 11 to 15, when the PV units inject more than 70% of their capacity, the voltage at node 63 exceeds the voltage at the slack node, reaching a maximum value of 1.0419 pu; while the minimum voltage value, as foreseen, is found at node 65, at hours 20 and 21, with a value of 0.9092 pu when the PV generation resource is not available.

### 5.3. Case 2: Results in the DC IEEE 33-Bus System

To demonstrate the versatility of the proposed DCVSA to solve the problem of the optimal siting and sizing PV sources in electrical networks, here, we present the application of this optimization method to the DC version of the IEEE 33-bus system reported in [53]. To obtain the DC version of this system, it is necessary to neglect all the reactive demand powers and reactances of the distribution lines. Regarding the operative technology, the electrical configuration of the DC equivalents is monopolar [54], i.e., where the voltage difference between a positive pole and a neutral cable is the same that is assigned for the AC grid.

Table 6 lists the comparison between the benchmark case and DCVSA solution. Numerical results specify that the proposed optimization method finds an objective function value of US$/year 2,662,425.32 by locating three PV generation units in nodes 9, 15, and 31. These generators have a total power installed capability of 3587.26 kWp, which permits a reduction of 26.94% with respect to the benchmark case, i.e., US$/year 981,617.69.

As per the simulation scenarios in the AC test feeders, to verify the effectiveness and robustness of the proposed optimization method in DC grids, we evaluate the DCVSA for the IEEE 33-bus system 100 consecutive times. The best response found was US$/year 2,662,425.32, a mean value of US$/year 2,664,496.59, and the worst result was US$2,667,733.661, with a standard deviation of US$/year 1652.82 (this represents a variation of 0.062% with respect to the average solution) and a processing time of 76.86 s. These results render it possible to analyze the following: (i) the proposed methodology is likely to generate the best average response or a response very close to this value in a radius of less than the US$/year 1700; (ii) if the worst solution given by the DCVSA is implemented, there will be a reduction of 26.79%, i.e., US$/year 973,547.57 when compared to the benchmark case; and (iii) the difference between the best and worst values is approximately US$8070.12 per year of operation, which is less than 0.25% of the annual operating cost in the benchmark case.

**Figure 10:**
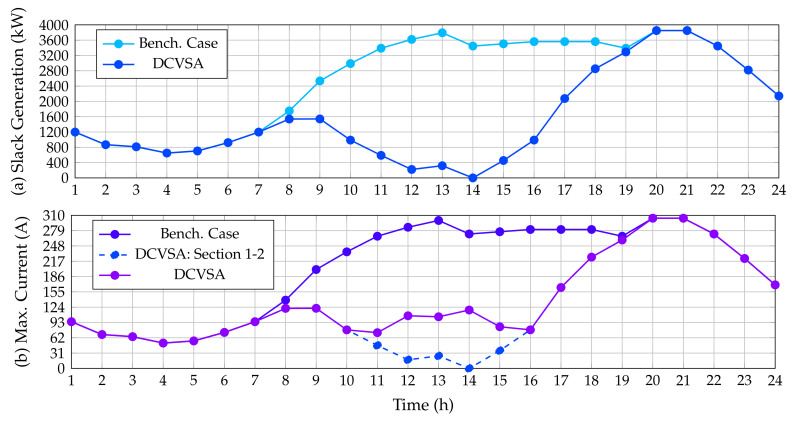
Impact of the PV inclusion in the DC version of the IEEE 33-bus system: (**a**) power injections in the slack source, and (**b**) maximum current performance.

Figure 10 presents the behavior of the power generation in the slack bus before and after the location of the PV generation sources; it also includes the current behavior of the system in both simulating conditions.

As anticipated, the generation in the slack node for the benchmark case follows the behavior of the demand curve measured in the substation terminals; however, when the PV generators are installed, the power output in the slack source is reduced considerably as the PV generation increases. Note that, in the period of time 14 when the renewable generation is maximum, the slack generation reaches a value of zero. It is worth mentioning that the slack generation curve in the DC version of the IEEE 33-bus system is about 75.74 kW lower than the AC case during the peak load scenario. This can be attributed to reactive power not flowing through the lines in the case of the DC grid, which implies that the level of energy losses in the DC grid equivalent is considerably minor than the AC grid as demonstrated in [55].

Figure 10 also presents the behavior of the maximum current flow in this test feeder. In the benchmark case, the maximum current flow occurs at the line that connects the nodes 1 and 2. This current exhibits the same performance of the power generation in the substation bus since it corresponds to the division between the output power and output voltage in this node; additionally, the maximum current value is found at periods 20 and 21 with a magnitude of 304.1278 A. However, after integrating the PV generation provided by the DCVSA, the maximum current that circulates through the system occurs in the network section 1–2 from hour 1 to 10 and from hour 16 to 24, since when the PV units supply more than 70% of their power, i.e., from the periods 11 to 15, the maximum current circulating through the system appears in the branch connecting the nodes 5 and 6. This occurs as there is no reactive power flow, and subsequently, the current in the network section 1–2 tends to follow the same behavior as the power generated in the slack node. Similarly, upon observing that the PV units are located in the two farthest branches of the system and considering the aforementioned situation, it seems reasonable that the greatest current is in the branch that connects nodes 5 and 6 since they join both branches at node 6.

**Figure 11:**
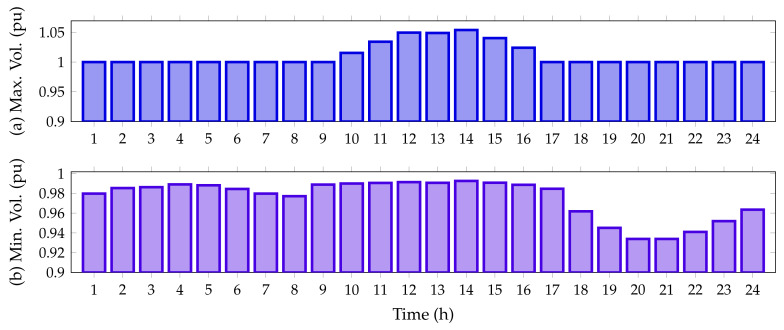
Voltage behavior during the day for the IEEE 33-bus system in its DC version: (**a**) maximum voltage magnitude and (**b**) minimum voltage magnitude.

On the other hand, Figure 11 details the minimum and maximum voltage values for the IEEE 33-node system in its DC version when all the PV generation units provided by the DCVSA are installed.

From Figure 11, it is observed that all the nodal voltages in this test feeder are between their minimum and maximum regulation bounds, i.e., ±10%. Additionally, from hours 11 to 15, when the PV units inject more than 70% of their capacity, the voltage at node 14 exceeds the voltage at slack node, reaching a maximum value of 1.0539 pu; while the minimum voltage value, as expected, is found at node 18, at hours 20 and 21, with a value of 0.9339 pu when the PV generation resource is not available.

### 5.4. Case 2: Results in the DC IEEE 69-Node System

This simulation case applies the proposed DCVSA to the IEEE 69-bus system in its DC version (details about the DC conversion of this test feeder are reported in [53]).

Table 7 showcases the optimal location and sizes of the PV generation units for this test feeder. The nodes selected were 23, 62, and 63, with an installed total generation capability of 3730.81 kWp. With these generation sources, the system’s objective function is US$/year 2,785,538.58, which corresponds to a reduction of 27.03% in the total grid operative costs, i.e., US$1,031,881.8 per year of operation with respect to the benchmark case.

It is worth mentioning that, after evaluating the DCVSA in this test feeder 100 consecutive times, the following results are determined: the best response was US$/year 2,785,538.58, the average solution value was US$/year 2,789,785.22, and the worst solution was US$/year 2,804,251.69, which has a standard deviation of US$/year 2710.94 and a processing time of 269.22 s. These results allow the observation of the following: (i) the proposed methodology is likely to generate the best average response or a response very close to this value in a radius of less than the US$/year 2800; (ii) if the worst solution given by DCVSA is implemented, there will be a reduction of 26.54%, i.e., US$/year 1,013,168.69 when compared to the benchmark case; and (iii) the difference between the best and worst values is approximately US$ 18,713.11 per year of operation, which is less than 0.49% of the annual operating cost in the benchmark case.

Figure 11 reveals the behavior of the active power generation in the slack bus for the benchmark case as well as for the solution provided by our proposed DCVSA. Additionally, this picture also presents the maximum current flow through the system for both simulation cases. As predetermined, the behavior of these variables in the IEEE 69-node test feeder is quite similar to the behavior analyzed in the IEEE 33-bus system. Note that the slack generation arrives at a value of zero when the generation in the PV units reached the maximum value (see hour 14); and the current in the branch that leaves the substation node is also zero in the same period since it is directly proportional to the power generation in the slack source. Moreover, the magnitude of the power generation in the slack source is reduced about 81.53 kW during the peak demand condition as a result of the nonexistence of the reactive power effects on the grid.

**Figure 12:**
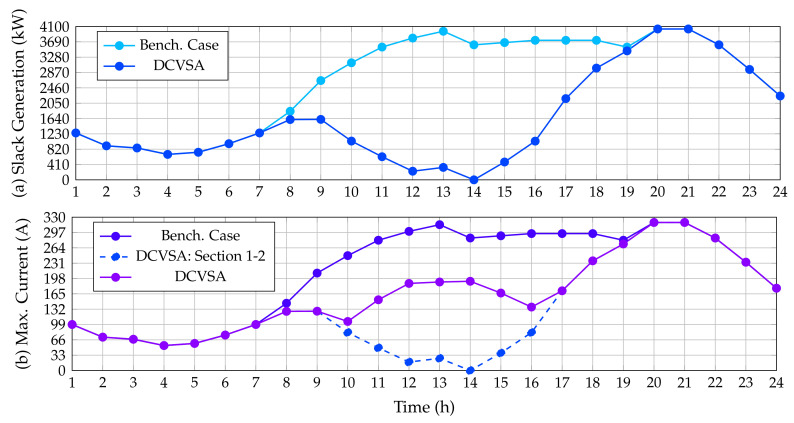
Impact of the PV inclusion in the DC version of the IEEE 69-bus system: (**a**) power injections in the slack source and (**b**) maximum current performance.

Additionally, Figure 12 presents the behavior of the maximum current in the IEEE 69-bus system in its DC version. Note that the maximum current expected in this system is equal for the benchmark case as well as for the DCVSA solution. The peak current occurs between the periods of time 20 and 21 when the demand power is maximum with a magnitude of 318.6598 A. It is worth noting that the branch current is maximum at the branch that connects nodes 1 and 2 from the hours of 1 to 9 and 17 to 24. However, due to the high penetration of the PV generation in the remaining periods, the maximum current appears in the branch connecting nodes 61 and 62; this is due to the location of the PV sources in nodes 61 and 63, which imply that, when the renewable generation output increases, the current in their area of influence will increase as well.

**Figure 13:**
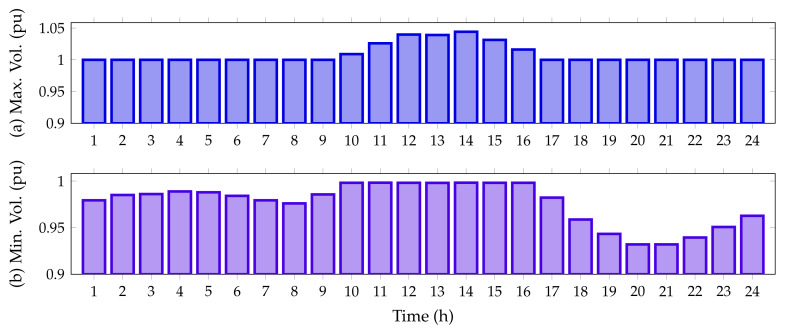
Voltage behavior during the day for the IEEE 69-bus system in its DC version: (**a**) maximum voltage magnitude, and (**b**) minimum voltage magnitude.

Figure 13 illustrates the upper and lower voltage magnitudes reached in the IEEE 69-bus system in its DC version when all the PV sources have been installed on this grid.

Note that, in the period of time 14, the voltage magnitude exceeds the voltage output at the slack source with a magnitude of 1.0442 pu. This appears at node 63, which has connected two PV generation units in its area of influence. Furthermore, as per the predicted variables, the worst voltage profile is experienced at node 65 with for the peak load condition (hours 20 and 21), where the PV generation availability is zero.

It is important to highlight that, for the DC versions of the test feeders studied, numerical validations were not presented with the comparative methods based on GAMS and DCCBGA implementations, since, as demonstrated in the AC simulation cases, the proposed DCVSA was highly superior with regard to the final objective function value. Moreover, in the current literature, there were no reports solving the analyzed problem in DC grids, which renders the selection of a fair comparative methodology to compare our proposed algorithm a difficult task.

## 6. Conclusions and Future Works

This research article presented a master–slave methodology to solve the location and dimensioning problem of PV systems in distribution networks through the application of the discrete-continuous version of the vortex search algorithm. In the master stage, the DCVSA was entrusted with defining the PV units’ optimal location and sizing, while in the slave stage, the value of the fitness function was determined using the matricial backward /forward power flow method. The objective function analyzed focuses on the minimization of the total annual operating costs which was composed of the annualized costs of purchasing energy at the substation node with the annualized investment costs for the PV units, including their maintenance costs. The numerical results demonstrated the applicability and efficiency of the optimization method developed for the studied test systems. The main findings are listed below.

✓ The reduction from the base case reached by DCVSA was 27.04%, and 27.15% for the test systems in their AC version; in their DC versions, the reductions were 26.94% and 27.03%, respectively.
✓ The proposed methodology obtained the lower standard deviation values when solving the PV units’ location and sizing problem for the IEEE 33- and IEEE 69-node test systems in their AC versions, with the values of US$/year 1154.08 and US$/year 2666.46, respectively. These values were considerably lower than the comparative DCCBGA, which confirmed the effectiveness and robustness of the proposed DCVSA to solve the studied problem ensuring that at each evaluation, the final objective function value will produce a small variation. In the case of the DC grids, these values were US$/year 1652.82 and US$/year 2710.94.
✓ Regarding the voltage profiles of both systems in their AC version, it was observed that, during the period of maximum PV energy injection, i.e., hour 14, the voltage at some nodes is above the voltage at the substation node, with the magnitudes 1.0291 pu and 1.0419 pu, respectively, while the minimum voltage values found during the time period of maximum power demand and minimum PV energy injection, i.e., in hours 20 and 21, had values of 0.9038 pu and 0.90919 pu, respectively. The same behavior was experienced in the DC grid equivalents. The most significant characteristic of these results is that it recorded evidence that the voltage regulation bounds assigned in ±10% of the nominal voltage were always fulfilled by the solutions reached by the DCVSA.
✓ The proposed solution methodology is independent of the number of nodes of the AC or DC network under study; however, in the number of nodes of the grid increase, the solution space size increases as well; this implies that the total processing times required to identify the optimal solution will also increase; however, these increments can be from a few minutes to hours, which is not a critical aspect in distribution system planning projects where the solution quality assumes the greatest importance instead of the total processing times.

For future works, it will be possible to examine and potentially solve the following: (i) solve the studied problem with new metaheuristic methods with high numerical performance as in the cases of the crow search algorithm, whale optimization algorithm, or black-widow algorithm, among others; (ii) extend the problem of the optimal location and sizing of PV generation units to bipolar DC grids with unbalanced operating characteristics; and (iii) formulate the problem studied in this research to three-phase distribution networks considering multiple constant power loads with Δ and *Y* connections.

## Figures and Tables

**Table 1:** Parametric information regarding the objective function calculation.

Parameter	Value	Unit	Parameter	Value	Unit
$C_{kWh}$	0.1390	US$/kWh	*T*	365	días
$t_{a}$	10	%	$N_{t}$	20	años
Δh	1	h	$t_{e}$	2	%
$C_{pv}$	1036.49	US$/kWp	$C_{0\&M}$	0.0019	US$/kWh
$N_{pv}^{ava}$	3	-	ΔV	±10	%
$s_{k}^{pv,\min}$	0	kW	$s_{k}^{pv,\max}$	2400	kW
$\alpha_{1}$	$100\times 10^{4}$	US$/V	$\alpha_{2}$	$100\times 10^{4}$	US$/V
$\alpha_{3}$	$100\times 10^{4}$	US$/W	$\alpha_{4}$	$100\times 10^{4}$	US$/A

**Table 2:** Numerical results in the AC version of the IEEE 33-bus system.

Method	Site and Size (Node, MVAr)	$A_{\mathrm{cost}}$ (US$/year)	$f_{1}$ (US$/year)	$f_{2}$ (US$/year)
Bench. Case	-	3,700,455.38	3,700,455.38	0
BONMIN	$\left\{17(1.35393),18(0.21051),33(2.14515)\right\}$	2,701,824.14	2,233,247.50	468,576.64
DCCBGA	$\left\{11(0.76046),15(0.96897),30(1.90598)\right\}$	2,699,932.29	2,240,724.98	459,207.31
DCVSA	$\left\{11(0.76061),14(1.08518),31(1.80295)\right\}$	2,699,761.71	2,238,872.09	460,889.62

**Table 3:** Numerical performance comparison between the DCVSA and DCCBGA in the IEEE 33-bus system after 100 consecutive evaluations.

Method	Best (US$/year)	Mean (US$/year)	Worst (US$/year)	SD (US$/year)	Avg. Time (s)
BONMIN	2,701,824.14	2,701,824.14	2,701,824.14	0	3.64
DCCBGA	2,699,932.29	2,702,178.35	2,705,870.99	1221.67	5.30
DCVSA	2,699,761.71	2,701,911.72	2,705,353.76	1154.08	170.23

**Table 4:** Numerical results in the AC version of the IEEE 69-bus system.

Method	Site and Size (Node, MVAr)	$A_{\mathrm{cost}}$ (US$/year)	$f_{1}$ (US$/year)	$f_{2}$ (US$/year)
Bench. Case	-	3,878,199.93	3,878,199.93	0
DCCBGA	$\left\{24(0.53255),61(1.89542),64(1.37716)\right\}$	2,825,783.32	2,345,138.38	480,644.95
DCVSA	$\left\{16(0.26321),61(2.27190),63(1.29335)\right\}$	2,825,261.56	2,341,670.47	483,591.08

**Table 5:** Numerical performance comparison between the DCVSA and DCCBGA in the IEEE 69-bus system after 100 consecutive evaluations.

Method	Best (US$/year)	Mean (US$/year)	Worst (US$/year)	SD (US$/year)	Avg. Time (s)
DCCBGA	2,825,783.32	2,829,498.36	2,844,469.50	2827.18	22.36
DCVSA	2,825,261.56	2,829,039.72	2,834,150.92	2666.56	887.64

**Table 6:** Numerical results in the DC version of the IEEE 33-bus system.

Method	Site and Size (Node, MVAr)	$A_{\mathrm{cost}}$ (US$/year)	$f_{1}$ (US$/year)	$f_{2}$ (US$/year)
Bench. Case	-	3,644,043.01	3,644,043.01	0
DCVSA	$\left\{9(0.58031),15(1.29137),31(1.71559)\right\}$	2,662,425.32	2,209,300.38	453,124.93

**Table 7:** Numerical results in the DC version of the IEEE 69-bus system.

Method	Site and Size (Node, MVAr)	$A_{\mathrm{cost}}$ (US$/year)	$f_{1}$ (US$/year)	$f_{2}$ (US$/year)
Bench. Case	-	3,817,420.38	3,817,420.38	0
DCVSA	$\left\{23(0.77201),62(2.34027),63(0.61853)\right\}$	2,785,538.58	2,314,281.30	471,257.28

## Data Availability

No new data were created or analyzed in this study. Data sharing is not applicable to this article.
